# Kindergarten teacher well-being: is bad stronger than good?

**DOI:** 10.3389/fpsyg.2025.1588793

**Published:** 2025-05-01

**Authors:** Markus Forster, Christof Kuhbandner

**Affiliations:** Departments of Human Sciences, University of Regensburg, Regensburg, Germany

**Keywords:** kindergarten teacher goals, kindergarten teacher emotions, kindergarten teacher well-being, negativity bias, undesirable behavior

## Abstract

Forster et al. demonstrated that school teachers’ well-being is related to their educational goals and experienced emotions for students showing undesirable behaviors: the higher the goals and the more positive the emotions, the higher the reported well-being. By contrast, the goals and emotions for students showing desirable behaviors was unrelated to school teachers’ well-being. These findings demonstrated that the principle of “bad is stronger than good” extends to the influence of student behavior on school teacher well-being. The present study examined whether this principle also applies to the well-being of kindergarten teachers who typically focus more strongly on the social–emotional development of children. We measured kindergarten teachers’ (*N* = 250) affective, evaluative, occupational, and psychological well-being using established questionnaires, and their educational goals and experienced emotions for children showing undesirable (e.g., children who provoke others, disrupt activities, cause physical harm) and desirable (e.g., children who share toys, comfort others, tidy up) behaviors using photorealistic pictures. Replicating the pattern observed for school teachers, the higher the goals and the more positive the emotions for children showing undesirable behaviors, the higher the well-being. By contrast, well-being was unrelated to the goals and the positivity of emotions for children showing desirable behaviors. However, the well-being of the kindergarten teachers was not completely unaffected by children showing desirable behaviors, as well-being was higher the higher the emotional arousal was in response to such children. These findings suggest that kindergarten teachers’ well-being could be improved by helping them to set high educational goals and experience more positive emotions for children showing undesirable behaviors, and to experience higher arousal for children showing desirable behaviors.

## 1 Introduction

Investment in early childhood education plays a critical role in various facets, particularly in children’s development (Sandilos et al., 2020) and economic sustainability (Heckman, 2006), while also helping to mitigate the risk of school burnout (Kleszczewska-Albińska, 2024). As a result, the well-being of kindergarten teachers is increasingly recognized as a crucial component in enhancing educational outcomes. Considering different types of well-being, that is, subjective (Diener, 1994; Diener et al., 1999) and psychological (Ryff and Keyes, 1995) well-being, existing research has shown that the well-being of kindergarten teachers is linked to a positive kindergarten climate (Tatalović Vorkapić and Velan, 2023), optimal functioning in the workplace (Benevene et al., 2018), collaboration among staff members (Miri et al., 2023), the quality of education provided to young children (Wong and Zhang, 2014), academic school readiness (Kotaman and Evran, 2023), the quality of teacher-child interactions (Penttinen et al., 2020), and their mindfulness and self-compassion (Leung et al., 2024). In addition, there are studies on promoting kindergarten teachers’ well-being through programs based on positive psychology (Armoza-Levi and Rusu, 2024) and mindfulness (Cheng et al., 2022). In summary, the well-being of kindergarten teachers is paramount not only for their own professional satisfaction but also for enhancing the overall quality of early childhood education.

In the recent years, research on kindergarten teachers has focused, among other things, on the effect of burnout on their mental state (for a review, see Fináncz et al., 2020), their challenges and innovative solutions (Akter and Hasan, 2024), teacher training programs (for reviews, see An and Zakaria, 2022; Eberhart et al., 2025), and coaching (for a review, see Yang et al., 2022). Surprisingly, although research into kindergarten teacher topics, and particularly their well-being has been intensified, it is still a topic that has been little researched (for reviews, see Cumming, 2017; Berger et al., 2022; Levi and Rusu, 2023). This is also due to the fact that studies on the well-being of teachers have tended to focus on teachers in primary and secondary schools (Benevene et al., 2018, for a review, see Hascher and Waber, 2021).

When comparing kindergarten teachers with other teachers, there are institutional differences regarding the age of children, teacher training and qualifications, as well as teamwork collaboration, the collaboration with parents, and specific challenges. The latter refer, for example, to the challenge faced in preschool children of differentiating between normal developmental variations and clinical disorders (Keenan and Shaw, 1997). Moreover, in early childhood education, there is a stronger focus on social and emotional development while teachers in primary and secondary schools often concentrate on academic content and exam preparation (Bennett, 2008). Furthermore, kindergarten teachers face significant emotional labor, yet research in this area remains limited (for a review, see Purper et al., 2023), especially when compared to studies involving teachers at higher levels of education. Addressing this research gap could provide valuable insights into how to support early childhood teachers and improve educational experiences for young children.

In a recent study, Forster et al. (2022) showed that school teachers’ well-being in secondary school is relatively strongly related to their goals and emotions for students showing undesirable behaviors: The higher the goals and the more positive the emotions, the higher the reported well-being. By contrast, the goals and emotions for students showing desirable behaviors were unrelated to school teachers’ well-being. These results demonstrated that the principle of “bad is stronger than good” (Baumeister et al., 2001, p. 323; for reviews, see Baumeister et al., 2001; Vaish et al., 2008) holds also for the influence of students’ behavior on the well-being of school teachers. Accordingly, it may be that also kindergarten teachers’ goals and emotions for children showing undesirable behaviors may contribute more to their well-being than those for children showing desirable behaviors.

The aim of the present study was to examine whether the results of Forster et al. (2022) can be replicated for the group of kindergarten teachers. In view of the differences between school teachers and kindergarten teachers described above, it is an open question whether the principle of “bad is stronger than good” can also be found in kindergarten teachers. To examine this question, we measured the goals and experienced emotions of kindergarten teachers for children showing desirable behaviors (e.g., children who share toys with others, comfort others, tidy up) and children showing undesirable behaviors (e.g., children who provoke and laugh at others, disrupt ongoing activities, hurt others physically), and determined the contributions of the kindergarten teachers’ goals and emotions for children showing desirable and undesirable behaviors to the kindergarten teachers’ affective, evaluative, psychological, and occupational well-being. Except for the fact that a different population (kindergarten teachers instead of school teachers) was examined, the original study was replicated as precisely as possible. The only main change was the use of new pictures depicting situations with kindergarten children instead of school.

## 2 Materials and methods

### 2.1 Participants

The study was preregistered (see https://osf.io/b67sv). In total, 250 German kindergarten teachers (240 women and 10 men) voluntarily participated in the study. There were no exclusion criteria. As indicated in the preregistration, data collection ended after 250 participants. Participants were mainly recruited by informing kindergarten teachers about the study and the possibility of voluntary participation, which was done by writing to kindergartens and via social media. Kindergartens across Bavaria were contacted until the desired sample size of 250 participants was achieved. To increase the motivation to participate and to minimize potential biases due to social desirability, all participants received automated personal feedback after the questionnaires for the study had been completed. The mean age was 42.15 years (ranging from 19 to 64 years, *SD* = 12.05), and on average, the participants have been working as a kindergarten teacher for 17.14 years (ranging from 0.6 to 44 years, *SD* = 11.39). Most of the participants worked full-time (60.0%) or part-time (38.4%), 66% in rural and 34% in urban areas. In Germany, young children are cared for in public daycare centers (KITAs), which are usually divided into two age-dependent facilities: Children aged one to two are cared for in nursery schools (German: “Kinderkrippe”), children aged three to six in kindergartens. Of the participants, 71.6% stated that they worked in kindergartens, 16.8% in nursery schools, and 11.6% in other types of day care centers. When asked about which age group they care for, 82.8% stated kindergarten and 17.2% nursery school. Reports on the professional qualification showed that early childhood educators was most frequent (*n* = 183; 73.2%), followed by child carers (*n* = 24; 9.6%), social pedagogues (*n* = 24; 9.6%), childhood pedagogues (*n* = 6; 2.4%), curative education nurse (*n* = 3; 1.2%), educational assistant (*n* = 3; 1.2%), trainee (*n* = 3; 1.2%), and other (*n* = 4; 1.6%).

The study was conducted in accordance with the Helsinki Declaration and the University Research Ethics Standards of the University of Regensburg. All participants provided written informed consent. In Germany, these types of psychological studies do not require ethical approval of an Ethics Committee (see https://www.dfg.de/foerderung/faq/geistes_sozialwissenschaften/).

### 2.2 Material and procedure

Self-report data were collected using the online platform SoSci Survey (Leiner, 2024). The study consisted of two phases. In the first phase, participants’ affective, evaluative, psychological, and occupational well-being was measured, using well-established questionnaires (see below). The sequence of questionnaires was the same for all participants. Directly afterwards, the second phase followed in which the participants’ goals and experienced emotions for children showing desirable behaviors and children showing undesirable behaviors were measured. Participants were instructed to put themselves mentally in the situation of a new kindergarten year where they will meet a new group of 16 children. They were told that the group will contain two types of children. It was emphasized that the distinction of two types of children is an oversimplification, and that in reality far more complex manifestations and mixed forms of these simplified types are found, and that we do not claim that there could be an ideal type. To avoid conceptual priming effects, the types of children were neutrally labeled as “type 1″ and “type 2″. The exact instruction was (original in German):

“A new kindergarten year: Please mentally put yourself in the situation that a new kindergarten year is beginning with a group of children you have not met before. On the next page, you will get to know your new children briefly, and perhaps you may have already encountered similar children in your real working life. To simplify the presentation, a distinction is made between two types of children. We are aware that in reality there are far more complex forms and hybrids of these simplified types, and we do not claim that there could be any kind of ideal type. Type I children are those who like to take part in group and individual activities and enjoy playing with the other children, that is, who usually show desirable behavior. Type II children are those who are less likely to take part in group and individual activities and less happy to play with the other children, that is, who usually show undesirable behavior.”

To introduce the group, the participants were first shown the eight pictures of the children showing desirable behaviors and second the eight pictures of the children showing undesirable behaviors. Each of the pictures was shown individually on the screen for 10 s each. In addition to the picture, a literal description of the behavior depicted in the picture was shown on the screen together with a fictitious name of the child. The development of the pictures of the two types of children was based on a study by Merrell (1996) where the preschool and kindergarten behavior scales (PKBS) were developed for screening and assessing social skills and social–emotional problems of young children. The social skills scale covers desirable behaviors (cluster description of behavioral characteristics: social cooperation, social interaction, social independence), the problem behavior scale covers undesirable behaviors (self-centered/explosive, attention problems/overactive, antisocial/aggressive, social withdrawal, anxiety/somatic problems). Based on these descriptions, eight photorealistic pictures of children showing desirable behaviors in a kindergarten situation and eight photorealistic pictures of children showing undesirable behaviors in a kindergarten situation were developed using chatgpt (OpenAI, 2023a,b,c) and DALL.E 3 (OpenAI, 2023a,b,c). To control for possible effects of gender, photographs of four male and four female children were used for each of the types. A detailed description of the pictures can be found at https://osf.io/r7cwf/files.

After the introduction of all children, participants’ educational goals were measured separately for children showing desirable and undesirable behaviors using an established questionnaire (see below). Before working on the respective questionnaires, all pictures of the children of the respective type were presented to the participants again together on the screen until participants pressed a button in order to start the questionnaire. After the questionnaire on educational goals had been completed, experienced emotions for the children were measured. Participants were shown the photographs of the 16 children individually on the screen for 5 s each in random order. After each presentation, the experienced emotions were measured using a combined version of the affect grid (Russell et al., 1989) and the self-assessment manikin (Bradley, 1994). There was no time limit for providing the respective emotional ratings.

### 2.3 Measures

#### 2.3.1 Affective well-being

Affective well-being was measured using the German version (Breyer and Bluemke, 2016) of the Positive and Negative Affect Schedule (PANAS; Watson et al., 1988), a self-report measure consisting of 10 positive (e.g., “enthusiastic”) and 10 negative (e.g., “distressed”) adjectives. Participants responded on a 5-point Likert scale ranging from 1 (not at all) to 5 (extremely) to describe how often they usually are in the respective emotional states. In the present sample, reliability on the 10 positive and negative items was high (Cronbach’s alphas = 0.87/0.87).

#### 2.3.2 Evaluative well-being

Evaluative well-being was measured using the German version (Glaesmer et al., 2011) of the Satisfaction With Life Scale (SWLS; Diener et al., 1985), a self-report measure consisting of five statements reflecting a positive evaluation of one’s life quality (e.g., “I am satisfied with my life.”). Participants responded on a 7-point Likert scale ranging from 1 (strongly disagree) to 7 (strongly agree). In the present sample, reliability was high (Cronbach’s alpha = 0.88).

#### 2.3.3 Psychological well-being

Psychological Well-Being was measured using the 18-item version of Ryff’s Psychological Well-Being Scale (PWB; Ryff et al., 2010), a self-report measure consisting of 18 statements reflecting the six areas of psychological well-being (the statements were adapted so that they referred to the context of kindergarten teaching): autonomy (e.g., “In my work at the kindergarten, I judge myself by what I think is important, not by the values that others think are important.”), environmental mastery (e.g., “I am good at handling the professional responsibilities of everyday life as a kindergarten teacher.”), personal growth (e.g., “I think it is important to have new experiences in my job that challenge how I think about myself and the world.”), positive relation with others (e.g., “At kindergarten, I am perceived as a giving person, willing to share my time with others.”), purpose in life (e.g., “Some kindergarten teachers wander aimlessly through life, but I am not one of them.”), and self-acceptance (e.g., “I like most parts of my personality regarding my work in kindergarten.”) The original 54-item version of Ryff’s PWB questionnaire (Ryff, 1989) has been translated into German by Risch et al. (2011), and the 18 items corresponding to the 18-item version of the questionnaire were used. Participants responded on a scale ranging from 1 (strongly disagree) to 7 (strongly agree). In the present sample, reliability was acceptable (Cronbach’s alpha = 0.72).

#### 2.3.4 Occupational well-being

Occupational well-being was measured using the job satisfaction scale of the Subjective Aspects of the Teaching Profession questionnaire (Dann et al., 2014), a self-report measure consisting of 12 statements (e.g., “I really enjoy my work as a kindergarten teacher,” “I am very satisfied with my job”). The statements were adapted so that they referred to the context of kindergarten teaching. Participants responded on a four-point Likert scale ranging from 1 (does not apply to me in any way) to 4 (applies to me completely). In the present sample, reliability was high (Cronbach’s alpha = 0.90).

#### 2.3.5 Educational goals

The participants’ educational goals for children showing desirable and undesirable behaviors were measured using an adapted version of the Questionnaire for the Assessment of Teacher Goals (FELZ; Rüprich and Urhahne, 2015), a self-report measure consisting of the scales of consideration of individual differences, student engagement, relationship with students, and learning impact, with four statements per scale. The statements were adapted so that they referred to the context of kindergarten teachers and to either the group of children showing desirable behaviors or the group of children showing undesirable behaviors (e.g., scale consideration of individual differences: “In my job as a kindergarten teacher, I strive to promote this type of children individually”; scale children engagement: “In my job as a kindergarten teacher, I strive to offer interesting activities for this type of children”; scale relationship with children: “In my job as a kindergarten teacher, I strive to build a trusting relationship with this type of children”; scale learning impact: “In my job as a kindergarten teacher, I strive to be a kindergarten teacher from whom this type of children develop further”). Participants responded on a five-point Likert scale ranging from 1 (I do not agree) to 5 (I agree completely). The total score was computed as the mean across all items. In the present sample, reliability was high (Cronbach’s alpha = 0.95).

#### 2.3.6 Experienced emotions

**Figure 1:**
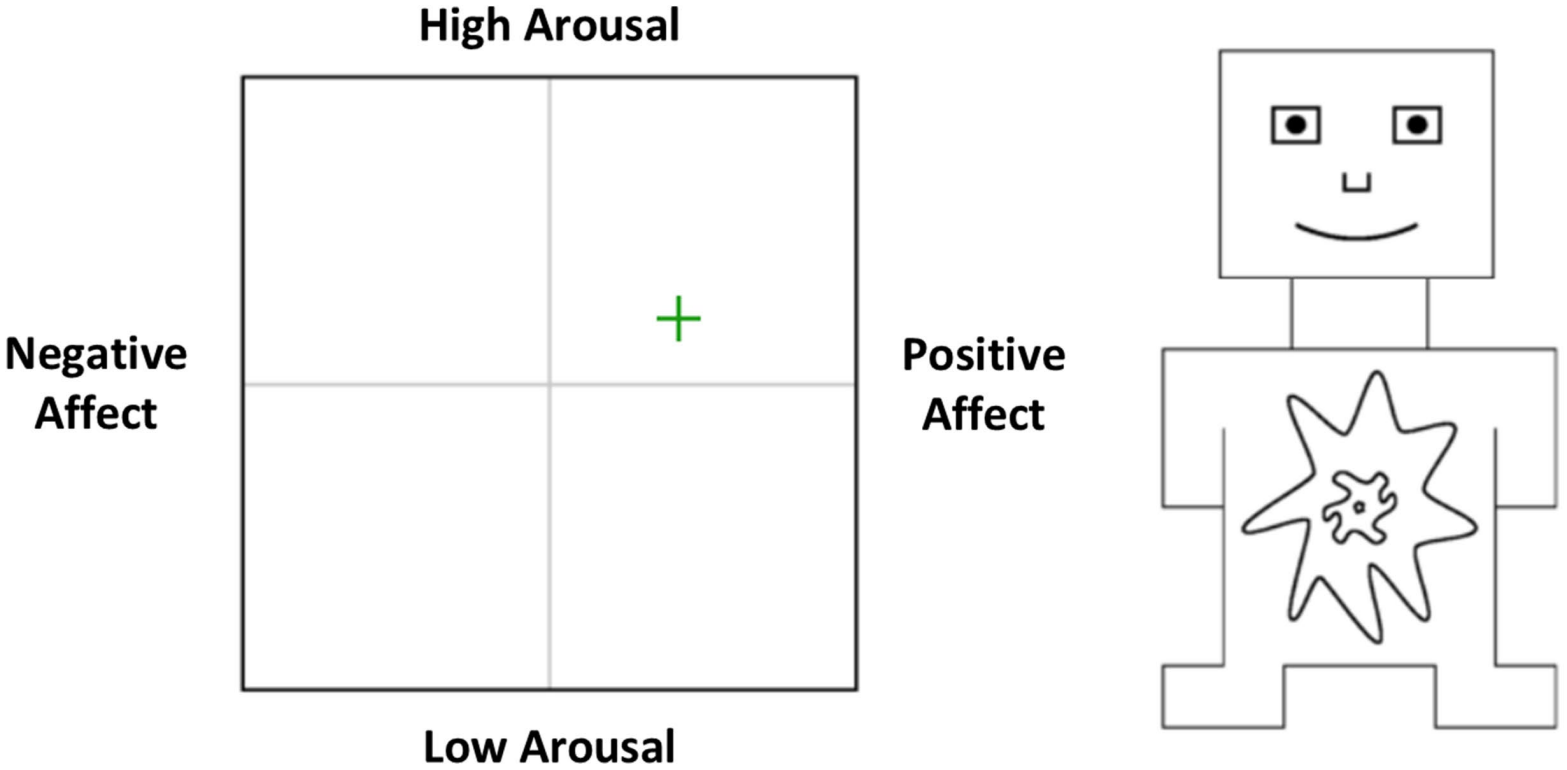
Illustration of the measurement of experienced emotions. To measure the experienced emotions, participants were presented with a so-called affect grid, which assesses experienced emotions on the dimensions of valence and arousal. Participants could move a cross across the grid. The further the cross was moved upwards, the more arousal was experienced; the further the cross was moved to the left, the more negative emotions were experienced; and the further it was moved to the right, the more positive emotions were experienced. To give the participants an intuitive idea of this type of emotion measurement, a small figure was shown next to it, modeled after the so-called Self-Assessment Manikin (Bradley, 1994), which dynamically changed its facial expression and the internal arousal level depicted on its body depending on the movement of the cross.

Experienced emotions were measured using a combined version of the affect grid (Russell et al., 1989) and the self-assessment manikin (Bradley, 1994). As depicted in Figure 1, an affect grid was shown on the screen which assesses experienced emotions on the dimensions of valence and arousal. Participants could move a cross across the grid, which resulted in respective changes in the manikin shown on the right side of the grid. That is, moving the cross along the valence axis changed the figure from frowning (negative) to smiling (positive), and moving the cross along the arousal axis changed the figure from eyes wide open and an explosive-like burst over the abdomen (high arousal) to eyes closed and a small pin prick over the abdomen (low arousal). The position of the cross on the grid on the valence dimension was converted in a valence score valence (−100 = extremely negative, +100 = extremely positive) and an arousal score (−100 = extremely low arousal, +100 = extremely high arousal). For both the pictures depicting children showing undesirable behaviors and those depicting children showing desirable behaviors, reliability was high (Cronbach’s alpha = 0.86/0.86).

## 3 Results

Table 1 shows the means and standard deviations for the measured variables as well as the correlations between all variables. Educational goals were higher for children showing desirable behaviors than for children showing undesirable behaviors, *t*(249) = 7.60, *p* < 0.001, *d* = 0.48. Experienced emotions were more negative and more arousing for children showing undesirable behaviors than for children showing desirable behaviors, *t*(249) = −35.64, *p* < 0.001, *d* = 2.25, and *t*(249) = 7.55, *p* < 0.001, *d* = 0.48. The correlational results show, on the one hand, that the different measures of well-being are moderately correlated with each other. On the other hand, the results show differential effects of educational goals and experienced emotions on well-being for children showing desirable behaviors versus undesirable behaviors.

**Table 1:** Correlations and descriptive statistics.

	1	2	3	4	5	6	7	8	9	10	11
1. Affective well-being (positive affect)		−0.33**	0.48**	0.52**	0.57**	0.03	0.10	0.01	0.05	0.20**	0.06
2. Affective well-being (negative affect)			−0.45**	−0.51**	−0.57**	−0.05	−0.04	0.07	−0.13*	−0.05	−0.01
3. Evaluative well-being				0.54**	0.55**	0.05	0.12	0.01	0.15*	0.15*	0.11
4. Psychological well-being					0.69**	0.16*	0.24**	0.08	0.14*	0.15*	0.17**
5. Occupational well-being						0.03	0.05	0.08	0.14*	0.17**	0.11
6. Goals (desirable behaviors)							0.69**	0.24**	−0.07	0.04	0.24**
7. Goals (undesirable behaviors)								0.27**	−0.07	0.02	0.27**
8. Experienced emotional valence (desirable behaviors)									−0.12	0.03	0.32**
9. Experienced emotional valence (undesirable behaviors)										0.21**	−0.02
10. Experienced emotional arousal (desirable behaviors)											0.03
11. Experienced emotional arousal (undesirable behaviors)											
*M*	3.533	1.78	5.18	5.42	2.88	4.47	4.65	60.92	−24.10	8.02	35.19
*SD*	0.65	0.65	1.12	0.61	0.62	0.50	0.39	20.70	29.12	44.73	36.51

*indicates *p* < 0.05, **indicates *p* < 0.01; *p*-values are not corrected for multiple testing. Significant correlations are printed in bold.

To examine the effects of educational goals and experienced emotions on well-being, multiple regression analyses were conducted with affective (positive and negative affect), evaluative, psychological, and occupational well-being as the dependent variables and (1) educational goals for children showing desirable and undesirable behaviors as independent variables, and (2) experienced emotions (valence and arousal) for children showing desirable and undesirable behaviors as independent variables. Results are shown in Table 2 (effects of educational goals) and Table 3 (effects of experienced emotional valence and arousal). Multicollinearity and heteroscedasticity diagnostics were conducted prior to the regression analyses. Correlations between predictor variables were all below *r* = 0.70, indicating no problematic bivariate collinearity. Furthermore, variance inflation factors (VIFs) were all below 5, and tolerance values exceeded the critical threshold of 0.20, suggesting that multicollinearity was not a concern. Visual inspection of residual plots did not reveal any clear patterns, and thus no evidence for heteroscedasticity was found.

**Table 2:** Results of regression analyses predicting level of affective, evaluative, psychological, and occupational well-being from kindergarten teachers’ educational goals for children showing desirable and undesirable behaviors.

Measure	*B*	95% CI		*SE B*	*β*	*t*	*p*
		*LL*	*UL*				
Affective well-being (positive affect; *R*^2^ = *0.01; R*^2^*_adj_* = 0.01)							
Goals (desirable behaviors)	−0.11	−0.33	0.12	0.12	−0.08	−0.91	0.362
Goals (undesirable behaviors)	0.25	−0.04	0.54	0.15	0.15	1.73	0.085
Affective well-being (negative affect; *R*^2^ = 0.00; *R*^2^*_adj_* = −0.01)							
Goals (desirable behaviors)	−0.07	−0.30	0.16	0.12	−0.05	−0.60	0.548
Goals (undesirable behaviors)	0.00	−0.29	0.29	0.15	0.00	0.00	0.997
Evaluative well-being (*R*^2^ = 0.02; *R*^2^*_adj_* = 0.01)							
Goals (desirable behaviors)	−0.17	−0.57	0.22	0.20	−0.08	−0.88	0.380
Goals (undesirable behaviors)	0.51	0.01	1.00	0.25	0.18	2.01	0.045
Psychological well-being (*R*^2^ = 0.06; *R*^2^*_adj_* = 0.05)							
Goals (desirable behaviors)	−0.02	−0.23	0.18	0.11	−0.02	−0.23	0.818
Goals (undesirable behaviors)	0.39	0.13	0.65	0.13	0.25	2.93	0.004
Occupational well-being (*R*^2^ = 0.00; *R*^2^*_adj_* = −0.01)							
Goals (desirable behaviors)	−0.03	−0.24	0.19	0.11	−0.02	−0.22	0.824
Goals (undesirable behaviors)	0.11	−0.17	0.38	0.14	0.07	0.75	0.453

CI, confidence interval; LL, lower limit; UL, upper limit; *β*, Standardized Regression coefficient; *t*, t-value; *p*, Probability of committing a Type-I-Error; *p*-values are not corrected for multiple testing.

**Table 3:** Results of regression analyses predicting levels of affective, evaluative, psychological, and occupational well-being from experienced emotional valence and arousal for children showing desirable and undesirable behaviors.

Measure	*B*	95% CI		*SE B*	*β*	*t*	*p*
		*LL*	*UL*				
Affective well-being (positive affect*; R*^2^ = 0.04; *R*^2^*_adj_* = 0.03)							
Experienced emotional valence (desirable behaviors)	0.000	−0.005	0.004	0.002	−0.01	−0.22	0.829
Experienced emotional valence (undesirable behaviors)	0.000	−0.003	0.003	0.001	0.01	0.16	0.871
Experienced emotional arousal (desirable behaviors)	0.003	0.001	0.005	0.001	0.20	3.06	0.002
Experienced emotional arousal (undesirable behaviors)	0.001	−0.001	0.003	0.001	0.06	0.96	0.337
Affective well-being (negative affect*; R*^2^ = 0.02; *R*^2^*_adj_* = 0.01)							
Experienced emotional valence (desirable behaviors)	0.002	−0.002	0.006	0.002	0.06	0.94	0.349
Experienced emotional valence (undesirable behaviors)	−0.003	−0.006	0.000	0.001	−0.12	−1.82	0.069
Experienced emotional arousal (desirable behaviors)	0.000	−0.002	0.001	0.001	−0.03	−0.47	0.641
Experienced emotional arousal (undesirable behaviors)	−0.001	−0.003	0.002	0.001	−0.03	−0.51	0.612
Evaluative well-being (*R*^2^ = 0.05; *R*^2^*_adj_* = 0.03)							
Experienced emotional valence (desirable behaviors)	−0.001	−0.008	0.006	0.004	−0.02	−0.23	0.820
Experienced emotional valence (undesirable behaviors)	0.005	0.000	0.010	0.002	0.12	1.93	0.054
Experienced emotional arousal (desirable behaviors)	0.003	0.000	0.006	0.002	0.12	1.88	0.062
Experienced emotional arousal (undesirable behaviors)	0.003	−0.001	0.007	0.002	0.11	1.71	0.088
Psychological well-being (*R*^2^ = 0.06; *R*^2^*_adj_* = 0.05)							
Experienced emotional valence (desirable behaviors)	0.001	−0.003	0.005	0.002	0.04	0.57	0.570
Experienced emotional valence (undesirable behaviors)	0.002	0.000	0.005	0.001	0.12	1.84	0.068
Experienced emotional arousal (desirable behaviors)	0.002	0.000	0.003	0.001	0.12	1.86	0.064
Experienced emotional arousal (undesirable behaviors)	0.003	0.000	0.005	0.001	0.15	2.33	0.021
Occupational well-being (*R*^2^ = 0.06; *R*^2^*_adj_* = 0.04)							
Experienced emotional valence (desirable behaviors)	0.002	−0.002	0.006	0.002	0.06	0.94	0.349
Experienced emotional valence (undesirable behaviors)	0.002	0.000	0.005	0.001	0.12	1.83	0.069
Experienced emotional arousal (desirable behaviors)	0.002	0.000	0.004	0.001	0.14	2.27	0.024
Experienced emotional arousal (undesirable behaviors)	0.001	−0.001	0.004	0.001	0.09	1.31	0.193

CI, confidence interval; LL, lower limit; UL, upper limit; *β*, Standardized Regression coefficient; *t*, t-value; *p*, Probability of committing a Type-I-Error; *p*-values are not corrected for multiple testing.

Regarding the relationship between well-being and educational goals, the results show that well-being depended solely on the height of educational goals for children showing undesirable behaviors while the height of educational goals for children showing desirable behaviors did not play any role (positive affective well-being: *β* = 0.15, *p* = 0.085; evaluative well-being: *β* = 0.18, *p* = 0.045; psychological well-being: *β* = 0.25, *p* = 0.004; all other *p*s > 0.36).

Regarding the relationship between well-being and experienced emotions, the results show that with regard to emotional valence, well-being solely depended on experienced emotional valence for children showing undesirable behaviors (negative affective well-being: *β* = −0.12, *p* = 0.069; evaluative well-being: *β* = 0.12, *p* = 0.054; psychological well-being: *β* = 0.12, *p* = 0.068; occupational well-being: *β* = 0.12, *p* = 0.069; all other *p*s > 0.348). With regard to emotional arousal, the results showed that well-being depended on emotional arousal for both children showing undesirable behaviors (evaluative well-being: *β* = 0.11 *p* = 0.088; psychological well-being: *β* = 0.15, *p* = 0.021) and desirable behaviors (positive affective well-being: *β* = 0.20, *p* = 0.002; evaluative well-being: *β* = 0.12 *p* = 0.062; psychological well-being: *β* = 0.12, *p* = 0.064; occupational well-being: *β* = 0.14, *p* = 0.024; all other *p*s > 0.348).

**Figure 2:**
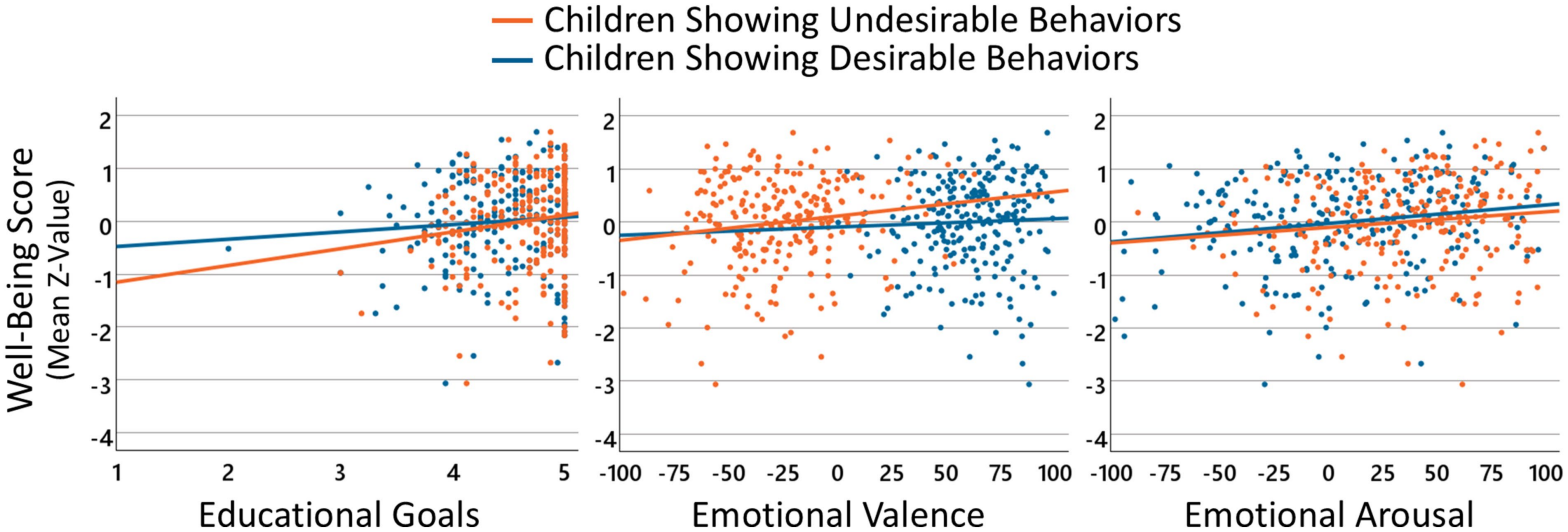
Relationship between kindergarten teachers’ well-being and educational goals and experienced emotions for children showing undesirable and desirable behaviors. The three panels show the relationship between the overall well-being score (averaged Z-values across affective, evaluative, psychological, and occupational well-being) and the level of educational goals pursued (left panel), the experienced emotional valence (middle panel) and the experienced emotional arousal (right panel) for children showing undesirable behaviors (orange color) and children showing desirable behaviors (blue color). Each dot represents the respective values of an individual participant, the lines depict the results of linear regressions.

Finally, for graphical visualization (see Figure 2), an overall well-being score was calculated across all types of well-being measured. For this purpose, following the suggestion that affective well-being represents the balance between one’s positive and negative affect (e.g., Diener and Ryan, 2009), first a value for affective well-being was determined by calculating the difference between positive and negative affectivity. All well-being scales were then z-transformed, and the mean of the Z-values was determined as the overall well-being score.

## 4 Discussion

Forster et al. (2022) provided correlational evidence that the principle of “bad is stronger than good” holds also for the influence of students’ behavior on school teachers’ well-being. The present findings indicate that this holds also true, for the well-being of kindergarten teachers. Well-being was the higher, the higher the educational goals and the more positive the experienced emotions for children showing undesirable behaviors. By contrast, well-being was unrelated to the goals and the positivity of the experienced emotions for children showing desirable behaviors. However, other than it was observed for school teachers, the well-being of kindergarten teachers was not completely unrelated to their emotional responses for children showing desirable behaviors because well-being was the higher the more emotional arousal was experienced for children showing desirable behaviors. In particular, the relationship between well-being and arousal was even slightly stronger for the arousal experienced for children showing desirable behaviors. While the arousal experienced for children showing undesirable behaviors was only related to evaluative and psychological well-being, the arousal experienced for children showing desirable behaviors was not only related to evaluative and psychological well-being but also to affective and occupational well-being.

The finding that kindergarten teachers’ educational goals and the positivity of experienced emotions for children showing undesirable behaviors are more strongly related with kindergarten teachers’ well-being than kindergarten teachers’ educational goals and the positivity of experienced emotions for children showing desirable behaviors are consistent with numerous studies showing for a broad range of psychical phenomena that bad events have more impact than good events (for reviews, see Baumeister et al., 2001; Vaish et al., 2008). The common explanation is that the higher psychological impact of bad events compared to good events is adaptive, as the costs of failing to adequately respond to bad events can be much higher than the costs of failing to respond appropriately to good events (Baumeister et al., 2001). Such an explanation could indeed also apply to the present findings. If undesirable behavior is overlooked and simply allowed to run its course, this can sometimes have very negative consequences for the children concerned, which must be prevented. In the case of desirable behavior, overlooking it and letting it run its course would not have any negative consequences; in fact, the opposite is the case. For this reason, it is also adaptive in educational contexts to focus more attention on undesirable behaviors than on desirable behaviors. And since attention resources are limited, desirable behaviors are perceived less strongly and can therefore have less influence on well-being. Such dynamics may be exacerbated by personality-specific influences. For example, people may tend to focus more on certain stimuli in their environment due to their previous emotional experiences, often prioritizing negative or information (e.g., Cisler and Koster, 2010). Furthermore, such tendencies could be reinforced by differences in emotion regulation, which may influence the way one responds to children showing undesirable behaviors. For instance, studies have shown that both instruction-induced state reappraisal and trait reappraisal are linked to reduced attentional bias to negative information (e.g., Kim et al., 2016).

However, the finding that the higher the emotional arousal for children showing desirable behaviors, the higher the well-being of kindergarten teachers, shows that the well-being of kindergarten teachers is not completely independent of children showing desirable behaviors. A possible explanation is that a stronger arousal in response to children showing desirable behaviors stems from a stronger activation of the approach system (e.g., Russell, 2003). In particular, such an explanation could also explain the differences in the role of emotional valence and arousal for children showing desirable versus undesirable behaviors. When interacting with children showing desirable behaviors, the approach system should generally be activated and the avoidance system deactivated, so that there is little variance in the emotional valence experienced, which would explain why there is no correlation between the experienced emotional valence for children showing desirable behaviors and well-being. The only thing that makes a difference in this case is how strongly the approach system is activated, which is reflected in the observed relationship between arousal and well-being. When interacting with children showing undesirable behaviors, both the avoidance system and the approach system can be activated, depending on the situation and the personality of a kindergarten teacher. A higher level of well-being would therefore occur when the approach system is more likely to be activated when interacting with children showing undesirable behaviors, which is reflected in the observed correlation between emotional valence and well-being. The smaller effect of arousal on well-being could then be explained by the fact that a stronger activation of the approach system can only have an effect on well-being in those cases in which the approach system is activated in response to children with undesirable behavior.

The relationships between goals, emotions, and well-being observed in the previous study on school teacher well-being (Forster et al., 2022) differ in several ways from those in the current study on kindergarten teacher well-being, which could have various causes. Overall, the relationship between well-being and educational goals and experienced emotions for children showing undesirable behaviors was weaker for kindergarten teachers compared to secondary teachers. One possible explanation could be that undesirable behaviors in children in the age group cared for by kindergarten teachers is interpreted less as disruptive and more as part of the spectrum of behavior observed in the normal development of children. In the latter case, undesirable behavior is not seen as a disruptive behavior to be avoided, but rather as an indication that the child needs to be approached and supported in order to master the developmental stage reached. At the level of educational goals, this has the consequence that higher educational goals are more likely to be set for children with undesirable behaviors, and at the level of experienced emotions, this has the consequence that undesirable behavior can be accompanied by an activation of the approach system at the level of the kindergarten teacher. Taken together, this would reduce the differences in responses to children showing desirable versus undesirable behaviors.

Another explanation could be that kindergarten teachers acquire higher competencies than school teachers during their professional training in the fields of developmental psychology and dealing with undesirable behaviors. On the one hand, this increases the ability to prevent and control undesirable behaviors, on the other hand, a more positive attitude toward children showing undesirable behaviors is promoted, which together reduces the differences in responses to children showing desirable versus undesirable behaviors as well. In this context, it is important to note that this study did not distinguish between undesirable behaviors that are part of normal development and those that arise from a background of disorders. Accordingly, it remains to be shown whether the observed relationships with the well-being of kindergarten teachers can be generalized across both types, an issue that should be explored in future studies.

The present study also provides possible approaches for promoting the well-being of kindergarten teachers, which should be examined in future studies. A first starting point arises from the finding that the goals and the positivity of the experienced emotions for children showing desirable behaviors did not play a significant role for well-being. Accordingly, one could try to support kindergarten teachers in placing more emphasis on the goals and emotions experienced for children showing desirable behaviors when assessing their well-being. One specific approach could be, for example, to practice the focusing of attention on children showing desirable behaviors in everyday working life, a technique that has already been proven to be beneficial for well-being in a variety of other areas (Knowles et al., 2016).

A second starting point arises from the finding that well-being was the higher, the higher the goals and the more positive the emotions for children showing undesirable behaviors. Accordingly, one could try to help kindergarten teachers to set higher educational goals for children showing undesirable behaviors, and to experience interactions with such children in a more emotionally positive way. One possible approach, as outlined in the previous paragraph, could be to convey the perspective that “undesirable” behavior at this age does not typically reflect “bad” intentions of the child, but rather the emergence of new developmental skills that require special guidance and support to develop. What could be further helpful would be the teaching of skills to better deal with undesirable behaviors, which would open up the space to pursue higher educational goals for children showing such behaviors. Helpful in this regard could be teaching kindergarten teachers strategies to better regulate triggered negative emotions, which has already proven to be very promising in the context of school teachers (e.g., Schelhorn et al., 2023).

Finally, in view of the finding that well-being was the higher the higher the experienced arousal, it could generally be helpful to achieve a high activation of the approach system when interacting with the children in care, for which it could in turn be helpful to strengthen the intrinsic motivation of kindergarten teachers (e.g., Deci et al., 1991).

Practical steps to enhance kindergarten teacher well-being could include interventions, trainings, and programs for educators in early childhood care, with a focus on promoting the abilities to regulate desirable and undesirable behaviors and emotions of children (Sutherland et al., 2018; Webster-Stratton, 2014) on the one hand, and to regulate one’s own emotions on the other hand (Schelhorn et al., 2023), which in turn can co-regulate children’s behaviors and emotions (Mänty et al., 2022; Parlakian, 2024). It is important to emphasize in this context that the well-being of kindergarten teachers is influenced by numerous other factors, many of which are not located at the individual level, but rather at the level of societal conditions, such as the availability of resources, the extent of collegial support, or the degree of societal appreciation (for a review, see Mukhlis et al., 2024). Since these factors define the scope for possible measures at the individual level, it is important, on the one hand, to take them into account when planning appropriate measures aimed at individuals. On the other hand, it is essential to work toward shaping these societal conditions in a way that optimally promotes the well-being of kindergarten teachers.

A limitation of this study, which is also relevant for considerations regarding the practical implications, is that the reported results only reflect correlational relationships between the variables. This means that the observed link between higher goals, more positive emotions, and better well-being in kindergarten teachers does not imply causality. For instance, it could be that teachers with higher well-being set higher goals or experience more positive emotions. The relationship could also be influenced by third variables. To establish causality, experimental designs manipulating goal-setting and emotions would be needed, such as a randomized controlled trial comparing teachers with and without targeted interventions on goal-setting and emotional experiences, which would be an interesting avenue for future research.

Another possible limitation of the present study could be that the behavioral goals and emotional responses were not measured in real life, but based on the presentation of pictures of children, in relation to which one should mentally imagine real professional life. Such a methodological approach has the advantage that the variables of interest can be better manipulated and potential confounding variables better controlled. However, this methodological strength comes at the cost of the fact that emotional responses may be stronger when elicited in real-life situations and may thus play a larger role for well-being as suggested by the measured emotional responses to pictures of kindergarten situations. In particular, viewing still images may not fully capture the dynamics of social interactions in real-life situations where kindergarten teachers have to concurrently address several behavioral patterns from several students. These limitations should be examined more closely in future research and taken into account when translating the findings of this study into practice.

Finally, it is important to emphasize that this study surveyed a sample of kindergarten teachers working in Germany. While the principle of ‘bad is stronger than good’ seems to be a fundamental, evolutionarily old principle of the human psyche, which is likely culture-invariant (e.g., Soroka et al., 2019), the effects of behaviors of children displaying desirable and undesirable behaviors on kindergarten teachers’ well-being are influenced by numerous contextual factors, which can vary across countries and cultures. These include factors such as group size, type and intensity of educational training, institutional support systems, and the interpretation of what behaviors are considered undesirable. Investigating the influence of such culturally varying contextual factors is an important question for future research.

In conclusion, the present findings indicate that the psychical principle of ‘bad is stronger than good’ seems also to apply to the influence of children’s behavior on kindergarten teachers’ well-being. Whereas educational goals and the positivity of experienced emotions for children showing undesirable behaviors had a comparably strong impact on kindergarten teachers’ well-being, their well-being was entirely independent of their goals and positive emotions for children showing desirable behaviors. Deviating from this principle, however, it was found that higher emotional arousal was particularly beneficial for well-being when interacting with children showing desirable behaviors. Taken together, a guiding principle for kindergarten teachers could therefore be: ‘Set high educational goals for children showing undesirable behaviors, keep aroused for children showing desirable behaviors, and do not lose the latter from your focus of attention’.

## Data Availability

The datasets presented in this study can be found in online repositories. The names of the repository/repositories and accession number(s) can be found in the article/supplementary material.
